# Imaging of ophthalmic manifestations: optical coherence tomography angiography and transorbital ultrasound in giant cell arteritis

**DOI:** 10.1007/s00296-025-05800-y

**Published:** 2025-02-11

**Authors:** Simon M. Petzinna, Jan H. Terheyden, Lara C. Burg, Claus-Juergen Bauer, Pantelis Karakostas, Charlotte Behning, Frank G. Holz, Robert P. Finger, Valentin S. Schäfer

**Affiliations:** 1 Department of Rheumatology and Clinical Immunology, Clinic of Internal Medicine III, University Hospital of Bonn, Bonn, Germany; 2 Department of Ophthalmology, University Hospital of Bonn, Bonn, Germany; 3 Institute of Medical Biometry, Informatics and Epidemiology, University Hospital of Bonn, Bonn, Germany; 4 Department of Rheumatology and Clinical Immunology, University Hospital of Bonn, Venusberg-Campus 1, 53127 Bonn, Germany

**Keywords:** Giant cell arteritis, Anterior ischemic optic neuropathy, Optical coherence tomography angiography, Ultrasound, Vascular ultrasound, Transorbital ultrasound

Dear Editor,

Giant cell arteritis (GCA) can lead to devastating ophthalmic complications, including sudden complete loss of vision due to anterior ischemic optic neuropathy (AION) secondary to inflammation and occlusion of the short posterior ciliary arteries, which supply the optic nerve head [1, 2]. Ischemia results in retinal changes, such as nerve fiber layer thickening, structural loss and optic disc edema [3, 4].

Assessing ophthalmic involvement in GCA remains challenging. Non-invasive optical coherence tomography angiography (OCTA) of the eye enables us to visualize the retinal and choroidal microcirculation [5, 6]. Additionally, we have recently shown that transorbital ultrasound (TOS) reveals changes in the ocular perfusion of symptomatic eyes in GCA patients [7]. Building on this cohort, we present the first study comparing vascular ultrasound, TOS, and OCTA findings in newly diagnosed GCA patients.

The study enrolled newly diagnosed and untreated GCA patients from the Rheumatology Department at the University Hospital Bonn, Germany. Diagnoses were made by a board-certified rheumatologist with patients further meeting the ACR-EULAR classification criteria [8] following ethics approval (ethics committee of the University Hospital Bonn, reference number: 097/18, october 2018). Demographic, clinical, and laboratory data were assessed. Each patient underwent a comprehensive ultrasound examination of superficial temporal, facial, axillary, carotid, and vertebral arteries, along with TOS using a Logiq S8 (2018; GE Healthcare; 6-15 MHz/ 8-18 MHz probes). Flow velocities of the central retinal artery (CRA), including peak systolic velocity (PSV), end diastolic velocity (EDV), resistance index (RI), and optic nerve diameter (OND) were assessed as described previously [7]. OCTA imaging was performed in one eye per participant, prioritizing symptomatic and right eyes. The OCTA protocol consisted of two 6 × 6 mm cube scans centered on the macula and optic disc (100,000 A-scans/second). OCTA outcome variables included vessel (VD) and skeleton density (SD), as well as vessel diameter index (VDI). Statistical analysis was conducted using SPSS version 26 and R version 4.3.0, with significance defined as *p*<0.05.

This study enrolled 23 patients, seven of whom presented with visual symptoms. Symptomatic eyes showed reduced PSV and EDV and increased OND compared to asymptomatic eyes (sTable 1). Overall, we found significant associations between ocular microcirculation in the superficial retinal plexus and the PSV in orbital ultrasound (Fig. 1). In the subgroup of patients with visual symptoms, the SD of the superficial plexus around the optic disc was positively associated with PSV on ultrasound (*r* = 0.83, *p* = 0.042). None of the OCTA variables capturing the deep capillary plexus were associated with any of the ultrasound parameters.

**Fig. 1:**
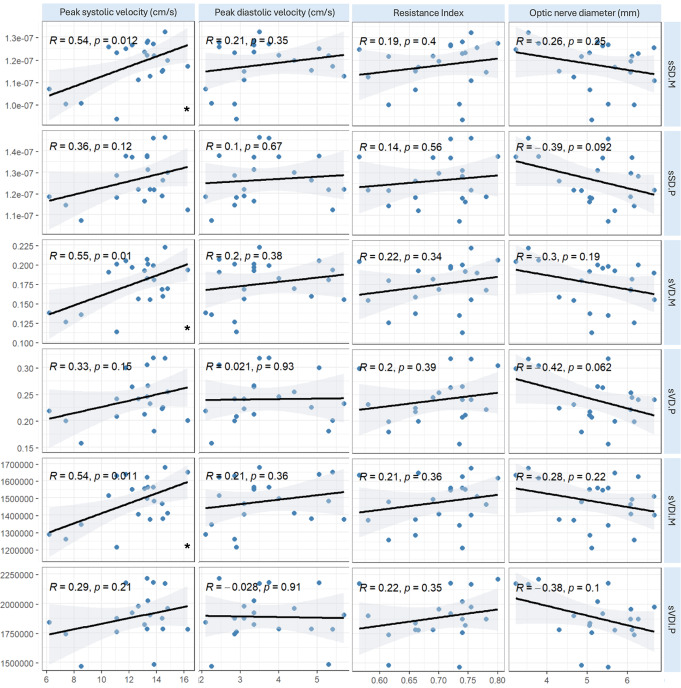
Correlation Plot Analysis of Optical Coherence Tomography Angiography Parameters and Transorbital Ultrasound in Newly Diagnosed Giant Cell Arteritis Patients. The figure exemplarily illustrates the results of correlation analysis conducted to evaluate association of the results of various parameters of optical coherence tomography angiography and transorbital ultrasound in newly diagnosed and untreated giant cell arteritis patients. Peak systolic velocity is shown to be significant positively correlated with superficial skeleton density and vessel density of the macula, with the correlation with other optical coherence tomography angiography parameters narrowly missing statistical significance. Abbrv.: s: supercifical, SD: skeleton density, M: macular, P: peripapillary, VD: vessel density, VDI: vessel diameter index; *p* < 0.05 was considered significant

Our study provides further insights into ophthalmic involvement in GCA. Expanding on our previous TOS study, which identified reduced CRA flow velocity and increased OND as biomarkers for ophthalmic involvement in GCA [7], we found significant positive associations between superficial OCTA parameters and PSV in symptomatic eyes. These results align with previous research showing that superficial retinal layers are commonly affected in AION [5, 6]. Notably, the associations between macroperfusion, as captured by ultrasound, and microperfusion, as captured by OCTA, were only moderate. This suggests prognostic relevance of both imaging modalities as they are complementary.

Interestingly, in contrast to previous studies indicating a greater impact of AION on the radial peripapillary nerve fiber layer [5], we found similarly strong associations of papillary and macular OCTA parameters with reduced CRA flow velocity, indicative of ocular GCA involvement. This was consistent across both symptomatic and all eyes, suggesting early ocular structural changes despite the absence of symptoms. This observation is further supported by the increased OND, which may indicate optic nerve head edema, and its negative association trend with superficial OCTA parameters, pointing to early retinal microvascular changes due to GCA.

In conclusion, this study is the first to compare vascular ultrasound, TOS, and OCTA findings in newly diagnosed GCA patients. We propose that analysis of both superficial and deep OCTA parameters, in conjunction with TOS imaging, should be performed, as it can provide complementary information for assessing ophthalmological involvement and ischemic damage in GCA. Future studies with larger cohorts and longitudinal follow-up are required to validate these findings and establish these imaging modalities as tools for monitoring disease activity and therapeutic response in GCA.

## Electronic supplementary material

Below is the link to the electronic supplementary material.

Supplementary Material 1

Supplementary Material 2

## Data Availability

The datasets collected for this manuscript are available from the corresponding author upon reasonable request.

## Declarations

### Patient and public involvement

Patients and/or the public were not involved in the design, or conduct, or reporting, or dissemination plans of this research.

### Disclaimer

This manuscript, including its text and graphics, is an original work and has not been copied or published elsewhere, in whole or in part. No artificial intelligence tools were used in the creation or writing of this manuscript.
