# Supplementary material for: Imaging of ophthalmic manifestations: optical coherence tomography angiography and transorbital ultrasound in giant cell arteritis
Source: Rheumatol Int. 2025 Feb 11;45(3):48. doi: 10.1007/s00296-025-05800-y (PMC11814037; doi:10.1007/s00296-025-05800-y)
Supplement: Supplementary file 2 — Supplementary Material 2 [file 296_2025_5800_MOESM2_ESM.docx]

**Supplementary Material**

**sTable 1:** Demographic, Clinical, and Laboratory Data and Results of Optical Coherence Tomography Angiography and Transorbital Ultrasound in Newly Diagnosed Giant Cell Arteritis Patients.The table presents the demographic, clinical, and laboratory data of newly diagnosed and untreated giant cell arteritis patients . Moreover, results of optical coherence tomography angiography and transorbital ultrasound are displayed dependent on their visual symptoms. Abbrv.: s: supercifical retinal capillary layer, d: deep retinal capillary layer, SD: skeleton density, M: macular, P: peripapillary, VD: vessel density, VDI: vessel diameter index

|  | **No visual symptoms (n = 16)** | | **Visual symptoms**  **(n = 7)** | | **Overall**  **(n = 23)** | |
| --- | --- | --- | --- | --- | --- | --- |
|  | **Mean** | **SD** | **Mean** | **SD** | **Mean** | **SD** |
| **Age (years)** | 76,3 | 8,4 | 72,9 | 9,7 | 75,3 | 8,76 |
| **Female (%)** | 50 | N/A | 57,1 | N/A | 52,2 | N/A |
| **Duration of symptoms (weeks)** | 15.8 | 18.5 | 9.31 | 8.46 | 13.8 | 16.2 |
| **Hb (g/dl)** | 12 | 1,28 | 12,4 | 1,09 | 12,2 | 1,22 |
| **Leucocytes (10^3^/ul)** | 9,16 | 2,22 | 10,4 | 2 | 9,52 | 2,19 |
| **Thrombocytes (G/l)** | 363 | 65,6 | 344 | 104 | 357 | 76,1 |
| **CRP (mg/l)** | 69,6 | 41,7 | 61,6 | 39,1 | 67,3 | 40,2 |
| **PSV (cm/s)** | 12,9 | 2,55 | 11,6 | 2,22 | 12,5 | 2,49 |
| **EDV (cm/s)** | 3,77 | 1,15 | 3,48 | 0,877 | 3,68 | 1,06 |
| **RI (PSV/ ED)** | 0,705 | 0,0606 | 0,691 | 0,0739 | 0,701 | 0,0635 |
| **OND (mm)** | 5,22 | 0,876 | 5,43 | 1,12 | 5,29 | 0,934 |
| **sVD.M** | 0,176 | 0,0263 | 0,174 | 0,0377 | 0,175 | 0,0296 |
| **sSD.M** | 1,18E-07 | 8,64E-09 | 1,17E-07 | 1,43E-08 | 1,18E-07 | 1,05E-08 |
| **sVDI.M** | 1.490.000 | 126.000 | 1.460.000 | 162.000 | 1.480.000 | 135.000 |
| **sVD.P** | 0,234 | 0,0431 | 0,256 | 0,0479 | 0,24 | 0,0446 |
| **sSD.P** | 1,25E-07 | 1,06E-08 | 1,31E-07 | 1,21E-08 | 1,26E-07 | 1,11E-08 |
| **sVDI.P** | 1.860.000 | 208.000 | 1.940.000 | 209.000 | 1.890.000 | 206.000 |
| **dVD.M** | 0,16 | 0,0251 | 0,162 | 0,0194 | 0,16 | 0,0229 |
| **dSD.M** | 1,29E-07 | 8,90E-09 | 1,30E-07 | 6,50E-09 | 1,30E-07 | 8,02E-09 |
| **dVDI.M** | 1.230.000 | 110.000 | 1.240.000 | 96.000 | 1.230.000 | 103.000 |
| **dVD.P** | 0,222 | 0,0453 | 0,218 | 0,0663 | 0,221 | 0,051 |
| **dSD.P** | 1,33E-07 | 8,57E-09 | 1,29E-07 | 1,27E-08 | 1,32E-07 | 9,84E-09 |
| **dVDI.P** | 1.680.000 | 352.000 | 1.670.000 | 421.000 | 1.680.000 | 363.000 |
